# Incorporating polygenic risk scores and social determinants of health across populations: Considerations and best practices in research

**DOI:** 10.1016/j.ajhg.2026.02.007

**Published:** 2026-03-04

**Authors:** Sara J. Cromer, Ewan K. Cobran, Hari S. Iyer, Micah R. Hysong, Luciana B. Vargas, Johanna L. Smith, Iain R. Konigsberg, David Bogumil, LáShauntá Glover, Gillian King, Polygenic Risk Methods Development (PRIMED) Consortium SDoH Working Group, Leslie A. Lange, Aniruddh Patel, Genevieve Wojcik, Laura Raffield, David V. Conti

**Affiliations:** 1 Diabetes Unit, Massachusetts General Hospital, Boston, MA, USA; 2 Department of Medicine, Harvard Medical School, Boston, MA, USA; 3 Programs in Metabolism and Medical & Population Genetics, The Broad Institute of MIT and Harvard, Cambridge, MA, USA; 4 Department of Quantitative Health Services, Mayo Clinic College of Medicine and Sciences, Scottsdale, AZ, USA; 5 Section of Cancer Epidemiology and Health Outcomes, Rutgers Cancer Institute, New Brunswick, NJ, USA; 6 Department of Genetics, School of Medicine, University of North Carolina at Chapel Hill, Chapel Hill, NC, USA; 7 Department of Biomedical Informatics, University of Colorado Anschutz Medical Campus, Aurora, CO, USA; 8 Cardiovascular Medicine, Mayo Clinic, Rochester, MN, USA; 9 Department of Population and Public Health Sciences, Keck School of Medicine of USC, Los Angeles, CA, USA; 10 Department of Population Health Sciences, Duke University School of Medicine, Durham, NC, USA; 11 Department of Epidemiology, School of Public Health, University of Colorado Anschutz Medical Campus, Aurora, CO, USA; 12 Mass General Brigham Heart and Vascular Institute, Massachusetts General Hospital, Boston, MA, USA; 13 Department of Epidemiology, Bloomberg School of Public Health, Johns Hopkins University, Baltimore, MD, USA; 14 Department of Biostatistics and Informatics, School of Public Health, University of Colorado Anschutz Medical Campus, Aurora, CO, USA

**Keywords:** polygenic risk, population descriptors, genetic ancestry, genetic similarity, race and ethnicity, social determinants of health, research ethics

## Abstract

There is a growing interest in evaluating the intersection of genetic and environmental factors, particularly social determinants of health (SDoH). As both the distributions and associations of genetic and SDoH-related risk vary across populations, a thorough understanding of the interplay of these factors (genetics and SDoH across populations) is necessary for the appropriate design and interpretation of studies examining their combined impact on health outcomes. In this review, we review population descriptors, including self-reported social constructs and genetically defined constructs, highlighting the different concepts they may capture and when it may be appropriate to use them. We discuss the challenges of applying polygenic risk scores (PRSs) to populations distinct in their genetic architecture or social context from the cohort in which they were developed. We provide an overview of conceptual SDoH frameworks and measures at the individual and area levels, discussing how these measures are defined, assessed, utilized, and interpreted in health research. For evaluating SDoH and PRS jointly, we outline analytic considerations, including calculating main-effect estimates, conducting gene-environment interaction studies, testing for mediation, and incorporating these factors into clinical prediction algorithms. When examining across populations, we highlight opportunities and challenges of data harmonization across existing cohorts and biobanks and ethical considerations necessary before embarking on or reporting work in this field. In all cases, we highlight the criticality of basing scientific questions upon well-considered conceptual frameworks arising from prior established relationships between risk factors and disease.

## Teaser

Despite increasing focus on the intersection of genetic and social risk factors on health, heterogeneity in how these factors are measured, distributed, and analyzed across populations and datasets complicates modeling efforts across contexts. We highlight complexities and best practices for research integrating genetic factors and social determinants of health.

## Introduction

The health and wellbeing of human populations are influenced by myriad factors that may be broadly classified into intrinsic (e.g., genetic) and extrinsic (e.g., environmental) categories. The genetic underpinnings of complex traits are increasingly modeled using polygenic risk scores (PRSs). These scores often use summary statistics from genome-wide association studies (GWASs) to create weighted scores quantifying an individual’s genetic risk for a trait or disease based on the totality of their genome rather than isolating single genetic variants.^1^ While myriad measures may be included in the “environment,” there is increasing recognition that social determinants of health (SDoH)—a diverse set of “conditions in which people are born, grow, work, live, worship, and age,”^2^—are an underlying driver of many psychosocial, cultural, behavioral, and physical environment exposures, with society- and population-specific effects.^3^^,^^4^ While molecular environmental exposures offer an expanded avenue of research to explore mechanisms and to enhance risk-prediction models, incorporation of each of these omic measures (such as methylation, metabolomics, or proteomics) brings additional study design, measurement, and analytic considerations.

Thus, an expanding field of research seeks to understand the intersection of complex trait genetics, SDoH, and clinical risk prediction across populations and how these features contribute to health disparities. Researchers are exploring ways to integrate these variables into biomedical research to better understand how SDoH interact with genetic predispositions. However, efforts to examine their combined effects are complicated by distinct distributions and performance of these measures across populations, correlation among these variables, underrepresentation of marginalized groups and informative missingness in many health datasets, inconsistencies in how population membership and SDoH are measured and categorized, and difficulty in identifying causal exposures. Moreover, these factors may interact in complex ways and mediate population-level differences in disease prevalence across racialized and ethnoracialized groups. The optimal means of measuring, analyzing, and interpreting analyses combining genetic and SDoH measures, particularly across diverse human populations, remains debated.^5^^,^^6^^,^^7^ Here, we aim to review and highlight the specific complexity that arises when incorporating SDoH measurements in clinical risk prediction, especially as they impact transferability of PRSs across populations and datasets.

This review aims to provide an overview of genetic risk prediction and SDoH concepts and measurement as they relate to analysis of health outcomes, with consideration of applications to diverse population groups. It outlines best practices for incorporating SDoH and environmental factors into genetic research while recognizing the complex interplay between genetic and environmental influences and how these relationships may differ across populations and contexts in both their distributions and effects. This review offers guidance for analyzing these variables in diverse populations, emphasizing the dynamic nature of the field; the need for ongoing refinement as understanding improves and community input expands; and the benefits of building interdisciplinary teams with expertise in the social sciences, population health, and community engagement.

**Figure 1:**
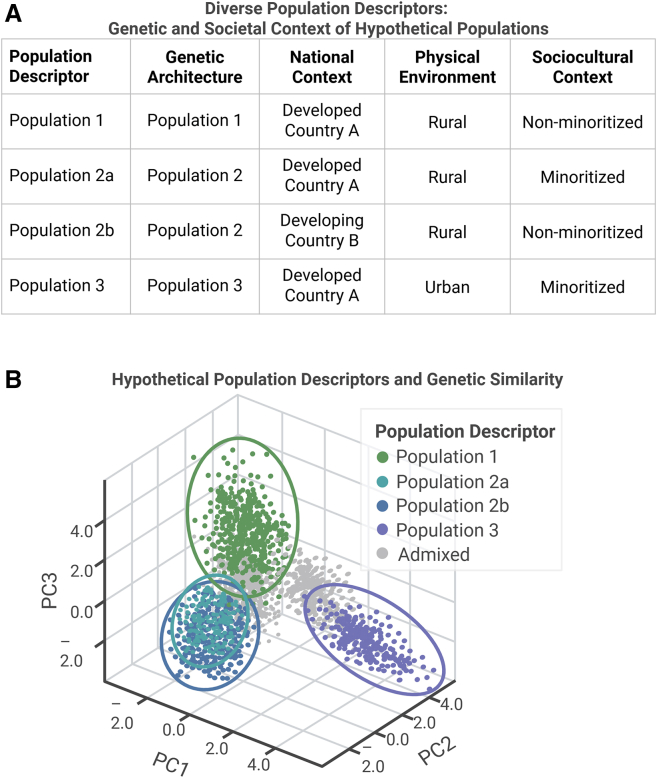
Population descriptors for hypothetical populations (A) In Figures 1, 2, 3, 4, and 5, we examine the complexities of modeling genetic and SDoH-related risk across populations using four hypothetical populations—each of which is racially and ethnically homogenous within its societal context—from four hypothetical datasets. Populations may be defined by diverse population descriptors, including those related to identity (e.g., race or ethnicity), lived experience (e.g., national or local environment, sociocultural context), or genetic relatedness (e.g., genetic ancestry or genetic similarity). (B) These hypothetical populations are defined by a combination of geography and self-identified race and have high intra-population genetic similarity but low inter-population genetic similarity. Note that many admixed individuals are not able to be categorized in any genetic ancestry population. PC = principal component. Created in https://BioRender.com.

**Figure 2:**
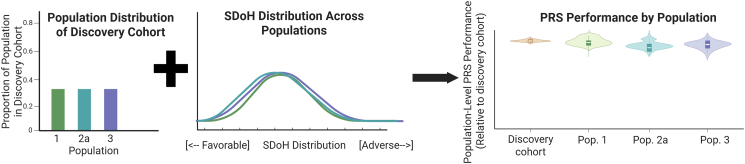
Idealized scenario with high PRS transferability across populations In an idealized scenario where PRSs are derived from GWAS in a discovery cohort representative of the study population and when the distribution and contextualization of potentially effect-modifying social determinants of health (SDoH) across populations are similar, the predictive performance of the PRS has the potential to be high and comparable in all study populations. Created in https://BioRender.com.

Throughout this review, we will highlight the complexities of genetic and SDoH-related analyses across populations through the lens of three hypothetical populations grouped by a combination of geography and racial identity, leading to high intra-population genetic similarity (as well as hypothetically homogeneous racial and ethnic identity within each populations societal context) but low inter-population genetic similarity (Figure 1). These differences in genetic architecture will have implications for the performance of PRS (Figure 2 v 3, path 1).^8^^,^^9^ In addition, population differences in geography and self-identified race and ethnicity will strongly associate with the SDoH to which these populations are exposed, including the patterns of SDoH factors that cluster together (Figure 4A), the distribution of adverse SDoH in the population (Figure 4B), the relationship between SDoH and study participation (i.e., selection bias; Figure 4B), and the magnitude and even direction of association of SDoH with disease in each societal context (Figure 4C). This heterogeneity in the clustering, distribution, and patterns of association of SDoH with disease will manifest as SDoH-population interactions affecting the distribution of health outcomes differently in each population (Figure 5).

**Figure 3:**
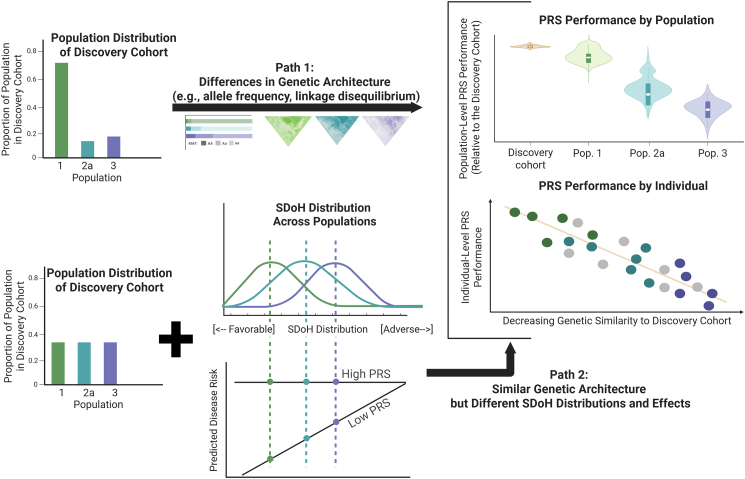
Common scenarios with reduced PRS transferability across populations PRS performance may decline at both the population and individual levels with decreasing genetic or SDoH-related similarity to the derivation population. This may occur if there is a lack of representation of all study populations in the discovery cohort, with distinct genetic architecture across populations in the application study (path 1) or if the study populations vary in the characteristics or contexts of their potentially effect-modifying SDoH (path 2; see Figure 4). Population-level PRS performance^9^ and individual-level PRS performance^8^ plots were adapted from published work. Created in https://BioRender.com.

**Figure 4:**
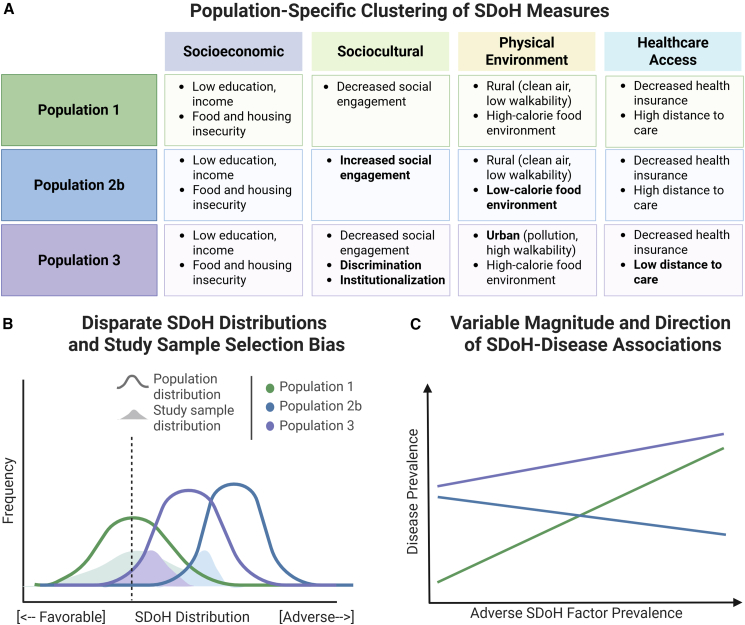
Considerations for SDoH application across populations SDoH may vary across populations in myriad, complex ways. (A) The clustering of SDoH measures may vary by population. In this example, adverse socioeconomic status is associated with some shared and some distinct sociocultural, physical-environment, and healthcare-access factors in each hypothetical population (bolded text highlights population-specific associations with low-socioeconomic status in each SDoH domain). (B) The absolute distribution of SDoH may vary substantially between populations, especially populations that do not share a broader societal context (e.g., from different countries). Here, population 2 and population 3 have more adverse and more narrow SDoH distributions than population 1 (mean SDoH indicated by dotted line; note that there is minimal overlap between the SDoH distributions of population 1 and population 2, which may limit the comparability of findings between these groups). More subtly, there may be differential selection bias across populations. Here, the included study sample for each population (shaded area) has similar SDoH distribution to the true underlying population for population 1 but has less adverse SDoH compared to the true underlying population for populations 2 and 3. (C) The magnitude and even direction of association of SDoH with disease may vary by population, with more adverse SDoH associated with increased T2D prevalence in populations 1 and 3 (albeit to different degrees) but with decreased T2D prevalence in population 2. This represents a population-by-SDoH multiplicative interaction or heterogeneity of SDoH effect (effect modification) by population. Created in https://BioRender.com.

As a concrete example of the complex interface of genetics and SDoH across populations, we examine a conceptual framework for type 2 diabetes (T2D) risk (Figure 6). T2D is a polygenic disease with high heritability^10^ as well as many environmental risk factors. It is also a highly heterogeneous disease that can result from multiple physiologic pathways relating to pancreatic β cell function and survival, overall and visceral adiposity, and organ-specific patterns of insulin resistance.^11^^,^^12^^,^^13^^,^^14^^,^^15^ Patterns of this heterogeneity have been noted to vary by racial and ethnic groups, with Black and Hispanic individuals in the US experiencing T2D onset at younger ages and with lower body mass index (BMI),^16^ and with East and South Asian individuals experiencing T2D onset both at lower BMI and with distinct patterns of insulin resistance and β cell deficiency compared to non-Hispanic White populations.^14^^,^^17^^,^^18^^,^^19^^,^^20^ Many PRSs exist for T2D, although most have been developed and have greater predictive accuracy in populations of European genetic ancestry (Figure 3, path 1).^21^ In addition to overall polygenic scores, “partitioned” polygenic scores (pPRSs) have also been developed to capture heterogeneity of physiologic pathways leading to T2D,^10^^,^^13^ and the distribution of these partitioned scores varies by genetic ancestry.^14^ Meanwhile, SDoH strongly associate with both T2D and one of its leading risk factors, obesity, and are differentially distributed across racial, ethnic, and genetic ancestry groups both within and between countries (Figures 4A and 4B). Further, the magnitude and even direction of the effect of SDoH on obesity and T2D vary by population (Figure 4C).^22^^,^^23^^,^^24^^,^^25^ Lastly, there are selection and information biases in many datasets that may differ by population. These include both selection bias and differential missingness in observational datasets resulting in decreased inclusion of marginalized groups and those experiencing adverse SDoH (Figure 4B).^26^^,^^27^^,^^28^ In addition, there may be differential misclassification of disease by SDoH and population group, with underdiagnosis of T2D in groups experiencing marginalization or more adverse SDoH.^29^ Racially and ethnically minoritized groups may experience structural and medical racism and present with “atypical” presentations of T2D compared to the majority White or European-ancestry populations best represented in medical research to date.

Given these considerations, it is expected that the definitions, distributions, and even effects of SDoH may vary across populations, and, when modeled as an interaction with a T2D PRS, which itself has variable distributions and performance across populations, analytic results may diverge in complex ways, requiring thoughtful and cautious interpretation. Stratified and sensitivity analyses examining for the effect of these differences in distribution, performance, and mechanisms leading to disease are also often necessary in order to explore this complex interplay.

## Population descriptors

As the distribution of PRSs and SDoH, their association with disease, and population-level disease prevalence all vary across populations, the intersection of these factors must be described within the contexts of the study population(s). While these ideas are discussed in detail elsewhere,^30^ here we summarize salient points for studies of PRS and SDoH across populations including the most commonly used contemporary population descriptors, the contexts in which they are used, and the historical and scientific context in which they were developed.

Human populations can be described and categorized by various characteristics, including geographic, demographic, and social factors, all of which have profound impacts on science, health, and society, particularly for historically minoritized groups. It is crucial to adopt best practices for respectfully and accurately describing populations in this evolving landscape.

The field of genetics has historically categorized participants using socially constructed descriptors (e.g., race and ethnicity) or more recently “descent-associated” descriptors (e.g., European or African continental ancestry) to reduce heterogeneity of genetic architecture within analysis groups. However, categorizations of both race and ethnicity and genetic ancestry rely on typological thinking, which imposes artificial and subjective divisions on the continuous spectrum of human diversity and may lead to the exclusion of individuals who do not fall into distinct categories (e.g., gray dots or “admixed” individuals in Figures 1B and 3), the perpetuation of health inequities, or the misconstrual of findings as supporting erroneous theories of racial essentialism.^31^^,^^32^ For example, T2D has a long history of racialization leading to both discrimination against groups believed to be more affected as well as perpetuation of misunderstandings about disease physiology.^33^ Recently, growing recognition of the fluidity of human experience and identity has led to increased focus on linear rather than categorical representations of populations,^30^ and expert groups now recommend use of genetic similarity, a relative and continuous measure that reflects how much DNA is shared between individuals or groups based on sampled genetic variation.^30^

### Race and ethnicity

While modern practice in the field of genetics is moving away from categorical descriptors, and specifically away from the use of societal constructs such as race and ethnicity, the use of race and ethnicity labels in health research, and their interpretation, remains both common and contentious.^34^^,^^35^ There are many examples where using such labels has hindered access to healthcare for minoritized groups^36^^,^^37^^,^^38^^,^^39^^,^^40^ but also evidence that failure to consider race and ethnicity may mask disparities.^41^ Medical and scientific institutions and journals therefore recommend that all academic authors and readers have a clear understanding of how these descriptors are defined and interpreted.^42^^,^^43^

Racial and ethnic classifications are socially constructed categories used to classify human diversity and are among the most widely available variables in research datasets.^44^ Race is typically assigned based on physical characteristics (e.g., skin color) or through self-identification with a particular group. In contrast, ethnicity refers to a group of individuals connected by shared cultural traditions, national origin, and language.^41^

As both are sociopolitical constructs without a basis in biology and with shifting definitions across time and context, individuals with similar appearance, genetic makeup, and cultural identities may self-identify or be assigned to different racial or ethnic categories in different societies, often based on categories imposed by majority groups that may not reflect the identities of affected populations.^45^^,^^46^^,^^47^ Similarly, the methods of capturing racial or ethnic identity in clinical research have evolved substantially over time, with growing preference for self-identification and both expansion and evolution of racial and ethnic categorizations in more modern datasets, leading to limitations in harmonization across datasets.^48^ As such, these alone should not be viewed as the sole explanation for inequities. Instead, race and ethnicity are understood to primarily act as proxy measures that generally influence health outcomes indirectly through factors linked to historical and contemporary drivers of inequality such as systemic and structural racism, which may manifest as disparities in the distribution of SDoH (Figure 4B).^35^^,^^49^^,^^50^

Analyses incorporating race and ethnicity therefore require thorough justification and rationale for use of these variables, development of robust causal frameworks linking race or ethnicity with outcomes through proposed causal intermediaries (such as SDoH or the lived experience of racism), and nuanced interpretation of study results so as not to exacerbate existing disparities in health. Whenever possible, the true exposure of interest should be measured and analyzed to avoid misinterpretation of race or ethnicity as causal determinants of disease and the perpetuation of racist stereotypes and practices.^51^ Such explicit frameworks ensure that the use of these variables is thoughtful and grounded in the study’s objectives and that the underlying causes of disparities are properly contextualized and understood rather than attributing differences solely to race.

### Genetic ancestry and genetic similarity

Genetic ancestry, in contrast, refers to the lineage of an individual’s DNA, tracing biological connections to shared ancestors. It is often categorized by continental origin in the current literature due to limited ability to assign more granular categories based on available reference panels and sample variation (e.g., as in Figure 1B).^52^ Numerous frameworks have been developed to ensure reproducibility of genetic ancestry designations in common datasets.^53^ However, with the growing recognition that human populations exist on a spectrum and the concomitant shift away from typological designations, expert guidance now recommends use of continuous measures of genetic similarity, rather than categorical genetic ancestry.^30^

Genetic similarity is a quantitative estimate of genetic relatedness between individuals, often expressed through principal components derived from genetic data, typically clustered with data from reference populations, or through mixture components from admixture analyses and related approaches.^54^^,^^55^^,^^56^ It is important to note, however, that commonly used measures of genetic similarity are influenced by the reference panels employed, with publicly available reference datasets often lacking adequate representation of certain groups, such as admixed Americans and East Africans.^52^^,^^57^^,^^58^ Unlike race and ethnicity or genetic ancestry, genetic similarity allows researchers to study human variation on a continuum. However, the operationalization of continuous genetic similarity in complex analyses remains a methodological challenge for interpretation when used as a confounder versus erroneously assigning risk to genetic similarity.

### Genetic risk prediction

Over the past decade, GWASs of complex traits have expanded rapidly, providing invaluable insights in biomedical research both by elucidating the molecular mechanisms underlying disease and by predicting genetic risk to inform epidemiological studies and preventive-medicine strategies.

To assess an individual’s genetic predisposition to common diseases, researchers often use PRSs derived from GWAS findings. In their simplest form, PRSs are individual-level scores that aggregate the number of risk alleles across the genome, each weighted by their effect sizes.^1^ Most predictive models in biomedical research aim to estimate the probability or age of onset of disease in asymptomatic individuals, with the ultimate goals of identifying high-risk individuals for targeted interventions, enabling more precise diagnoses, or predicting outcomes to therapeutic interventions. More recently, clustering methods have also been employed to explore genetic and phenotypic heterogeneity of disease, generating partitioned polygenic scores that may capture unique physiologic pathways to disease in both individuals and populations (Figure 6).^10^^,^^13^^,^^14^ These approaches hold great potential for personalized healthcare, offering tailored prevention and treatment strategies.^59^ However, the predictive accuracy of PRS-based models is limited by the heritability of the phenotype, which may vary between populations, due to differences in genetic architecture or the degree to which non-genetic factors contribute to the phenotype. In particular, population-level differences in allele frequency and linkage disequilibrium, disease-specific differences in patterns of inheritance and polygenicity (e.g., number of causal variants, common vs. rare variants), between-population variability in environmental risk factors, and gene-gene or gene-environment interactions may impact PRS performance (Figure 3). For example, if the overall variance in non-genetic factors is high, the genetic heritability may appear lower in that population.

### Transferability of PRSs across population and SDoH contexts

PRSs are typically based on common genetic variants with a minor allele frequency (MAF) of at least 1% that are likely to have emerged early in human history and are therefore expected to be shared across populations. The majority of contemporary PRSs have been constructed using data from Eurocentric GWASs, and the predictive accuracy of these scores declines significantly in populations that are genetically distinct from the discovery population, including those with complex genetic backgrounds due to recent admixture.^1^^,^^60^^,^^61^^,^^62^ For example, when applying European-based GWAS data, the accuracy of PRSs in African-ancestry populations is often only 20%–40% of that observed in European populations.^9^^,^^63^ Without efforts to enhance the generalizability of PRSs across diverse populations, applying current models may exacerbate existing health disparities.

To illustrate this, we examine three hypothetical populations grouped by a combination of geography and racial identity, leading to high intra-population genetic similarity but low inter-population genetic similarity (Figure 1). A hypothetical PRS derived from a GWAS including individuals of diverse genetic ancestry and similar distributions of SDoH would have good performance across hypothetical populations 1–3 (Figure 2). However, in practice, most PRSs have limited transportability due either to poor representation of some populations in the discovery cohort or distinct patterns of SDoH^64^^,^^65^ in the discovery and application cohorts, resulting in decreasing marginal effects of the PRS in other populations (Figure 3).

Recent theoretical and empirical studies have aimed to disentangle the factors influencing the transferability of PRS across diverse populations.^59^ While these generally suggest that the allelic effects of causal variants are consistent across ancestries,^66^ reduced PRS accuracy is primarily driven by differences in allele frequencies and linkage disequilibrium (LD) structures, with factors like fine-scale population structures, variability in the portability of genetic effects, and differences in non-genetic cohort characteristics between discovery and target populations also contributing.^1^^,^^67^^,^^68^^,^^69^^,^^70^ Improving PRS accuracy in African-ancestry populations is particularly challenging due to the limited representation of their greater genetic diversity in current genomic studies and reference panels.^71^ These challenges have led to increased efforts in data generation and methods development aimed at enhancing PRS accuracy across ancestrally diverse populations.^72^^,^^73^ A full accounting of the genetic architecture across the phenotypic spectrum and diverse ancestries, and its impact on the transferability of PRSs, requires continued expansion of non-European genomic resources and a comprehensive catalog of global genetic and phenotypic variation.^59^

While initiatives work to expand genetic data from historically underrepresented groups, simultaneous efforts are also underway to improve the transferability of existing PRSs across diverse populations. Several methods now aim to account for population- or ancestry-specific allele frequencies and effect sizes. One common approach is to combine GWASs from multiple ancestries using fixed-effect meta-analysis, followed by PRS construction based on the meta-GWAS results and a single-population PRS method.^59^ This technique, frequently used in large-scale multi-ancestry GWASs, enhances PRS transferability compared to population-specific scores.^74^^,^^75^^,^^76^ However, it poses challenges related to mixed LD patterns, may limit accuracy by assuming homogeneous allelic effects across populations, and may reduce both predictive value and transferability by restricting meta-analysis to variants common across populations.^59^ Alternative approaches leverage large-scale GWASs, often from European populations (referred to as the “auxiliary GWAS”), to improve prediction in a target non-European population with smaller GWASs. This methodology relies on the observations that many causal variants are shared across populations, and genetic correlations for complex traits and diseases are often moderate to high across ancestries.^77^^,^^78^^,^^79^ Moreover, multiple methods have recently been developed to improve PRS accuracy in admixed individuals by modeling ancestry-differential effects by leveraging local ancestry information.^59^^,^^79^^,^^80^^,^^81^

Recent research has also shown that social and environmental factors may influence the performance of PRSs across different populations and societal contexts,^68^ possibly due to selection bias of research participants, differences in the distribution of risk factors, or gene-environment interactions (Figure 3, path 2; Figure 4). However, their impact remains less understood. A goal of this work is to focus on the potential implications of SDoH on PRS transferability across populations.

### SDoH

#### The scope of SDoH: Contexts, domains, and levels of measurement

**Figure 7:**
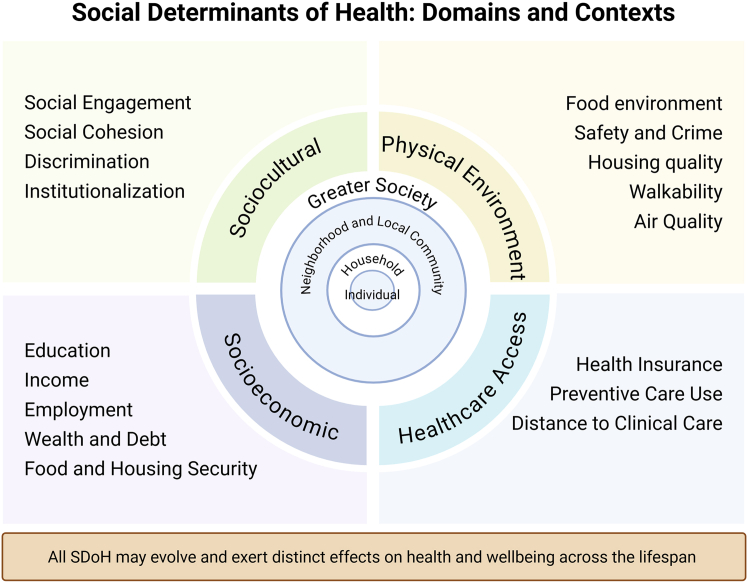
Framework for understanding SDoH SDoH are often categorized into four or more domains, each acting across social contexts, from the individual to the greater society, and across the lifespan. Within each of these domains, diverse measures exist to assess SDoH at either the individual or area level. Created in https://BioRender.com.

SDoH are the “conditions in the environments where people are born, live, learn, work, play, worship, and age that affect a wide range of health, functioning, and quality-of-life outcomes and risks.”^2^ Numerous conceptual frameworks exist to help researchers contextualize SDoH and categorize environmental exposures, often organized into SDoH contexts, domains, and levels of measurement (Figure 7),^2^^,^^82^^,^^83^ with each element of SDoH having the ability to change frequently throughout the lifespan and with SDoH exerting different effects on health and wellbeing based on the timing or stage of life of the exposure. This organization reflects theories posed by population scientists that the interventions required to narrow population-level differences in health are distinct from those delivered to individuals.^84^^,^^85^ Ecological models seek to characterize the influence of these SDoH on each of these contexts: the individual, the family and other interpersonal relationships, the local community or neighborhood environment, and the wider society. For each of these contexts, a variety of SDoH domains are recognized and broadly categorized into (1) socioeconomic factors, (2) sociocultural factors, (3) physical environmental factors, and (4) healthcare and healthcare-access-related factors.

These are the four SDoH domains on which this report is focused. While some experts also consider behavioral or lifestyle factors^82^—such as diet, smoking, sleep quality, and certain biological factors (e.g., the microbiome, metabolomics, or small molecules such as heavy metals; known as the “internal exposome”)^86^—to be SDoH due to their environmental and sociocultural influences, this classification is less consistent. In this review, these behaviors and biological factors will not be considered as SDoH given that they are heterogeneous in their etiology, resulting from both internal and external exposures, and in some cases may even be consequences, rather than causes, of underlying disease.^87^

For each of these domains, SDoH may be measured at either the individual (e.g., years of educational attainment) or area (e.g., ZIP code-level percentage with a college degree) level, reflecting different mechanisms of SDoH exposure for a given individual. Individual-level measures are usually acquired through surveys (interviewers or participant self-report), in which participants are asked about themselves and their lived experiences. By contrast, area-level measures of SDoH are often captured in large administrative databases such as the American Community Survey and assessed at different spatial scales. These measures consider how community-level factors—such as local COVID-19 prevalence, neighborhood socioeconomic status (SES), or environmental exposures such as air pollution—affect individual health outcomes.^88^ In general, individual-level SDoH capture more proximate risk factors and richer variability, and they may more precisely capture the specific environmental or social factor of interest for an individual; however, multi-faceted individual-level SDoH may not be available within or across datasets, whereas area-level SDoH are readily available in datasets with granular geographic information, are more easily harmonized across studies, and capture unique contextual and systemic factors contributing to individual risk. In studies with access to both individual- and area-level SDoH, the inclusion of both factors may most accurately capture an individual’s personal and societal context.^82^^,^^83^^,^^89^^,^^90^^,^^91^ Aside from individual and area levels of measurement, SDoH can also be assessed contemporaneously or for a particular stage of life (e.g., childhood socioeconomic or sociocultural context).

**Figure 5:**
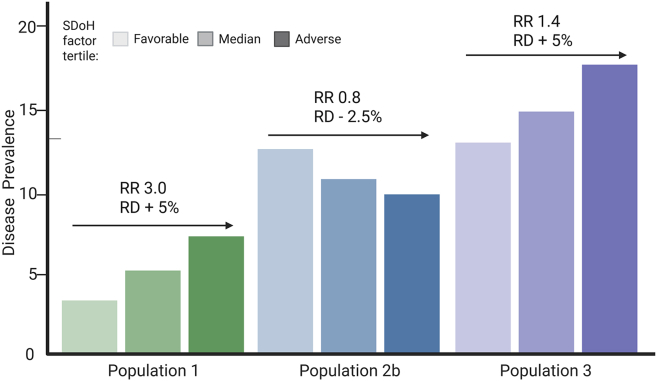
Additive and multiplicative interactions of SDoH with populations Related to the heterogeneity of SDoH across populations as depicted in Figure 4, the prevalence of T2D may vary significantly by population. In this example, population 3 experiences a greater than 3-fold increase in overall prevalence of T2D compared to population 1. Additionally, due to the heterogeneity of SDoH associations with T2D (Figure 4C), the prevalence of T2D at different SDoH levels, and thus the relative and absolute differences in disease prevalence across levels of SDoH, also differs by population. For example, there is an interaction on the relative scale between populations 1 and 3 (relative risk 3.0 for population 1 vs. population 3) but not on the absolute scale (risk difference 5% for both populations); population 2 demonstrates interactions on both the relative and absolute scales compared to either population 1 or population 3. RR, relative risk; RD, risk difference. Created in https://BioRender.com.

**Figure 6:**
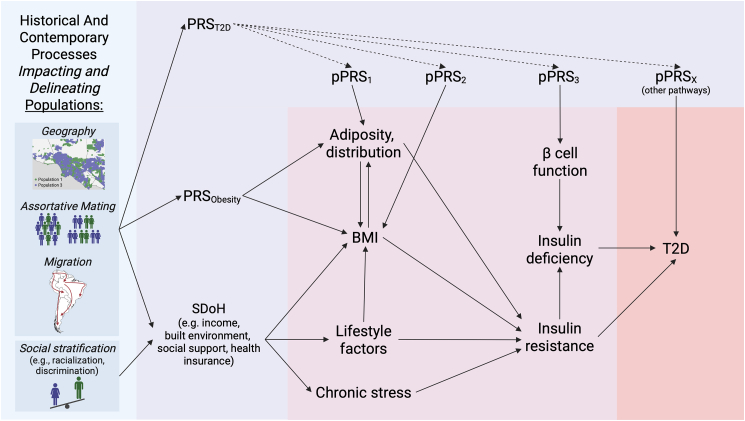
A conceptual framework incorporating genetic and SDoH-related risk for T2D across populations T2D risk is attributable to a complex interplay of genetic and environmental factors, both of which are related to SDoH through either upstream historical and contemporary processes (e.g., geography, migration, assortative mating, discriminatory practices) creating relationships between genetic architecture and SDoH, often through societal constructs delineating populations such as race and racism or through downstream effects by which associations between PRS or SDoH and T2D are modified or mediated by environmental and lifestyle factors. Due to the population-level differences in PRS performance, possible differences in prevalence of partitioned polygenic risk for specific T2D-causing pathways, unique clustering and distribution of SDoH, and overall prevalence of diabetes, the primary pathways leading to T2D may also vary by population. Note that not all possible factors or direct and indirect paths are shown, and PRS performance and the relative association of SDoH with disease may vary across populations (see Figures 3 and 4). Created in https://BioRender.com. pPRS, partitioned polygenic score; BMI, body mass index.

No gold standard has been established for measuring SDoH within or between populations. As such, researchers must determine, based on their question of interest, what the most appropriate SDoH context, domain(s), and level(s) of measurement may be, depending on study-specific factors including the hypothesized causal framework of the study, data availability, and the study question at hand. In many cases, this decision may be both population and disease specific. The hypothetical populations outlined in Figure 1A have distinct patterns of SDoH clustering, distributions, and associations with disease (Figure 4) such that use of a single SDoH measure (e.g., poverty) may capture different SDoH risk profiles and result in distinct patterns associations with disease prevalence in each population (Figure 5). Using the example of T2D, certain SDoH factors—namely those affecting an individual’s food environment, activity level, chronic stress, and access to healthcare (Figure 6)—will have larger impacts on disease outcomes compared with other factors (e.g., air pollution, which may alternatively be highly relevant for respiratory diseases). However, the clustering of these factors with other SDoH measures may lead to counterintuitive associations with disease (for example, resulting in a seemingly protective effect of poverty in population 2; Figures 4C and 5).

This section provides an overview of key SDoH domains at both individual and area levels of measurement (Table 1), offering practical strategies for integrating these factors into genetic epidemiological research studies. Acknowledging our primarily genetics-focused background, we emphasize the importance of fostering interdisciplinary collaborations with sociologists and health equity experts as our research into this intersectionality continues to evolve and expand. Below, we summarize how individual and area-level SDoH are commonly categorized, defined, and measured.

**Table 1:** Domains and commonly assessed measures of SDoH at the individual or area level

**Domain**	**Common individual-level measures**	**Common area-level measures**
Socioeconomic status	• educational attainment • income • percentage of poverty threshold • employment • net worth • receipt of public assistance • home or car ownership • food or housing security • debt	• percentage in [area] with(out) HS degree • percentage in [area] with(out) college degree • median household income • median income-to-poverty ratio • percentage living below a poverty threshold • median home value
Sociocultural	• family and social group cohesion • caregiver burden • social engagement (e.g., Social Network Index)^92^ • experiences or perception of discrimination • acculturation,^93^ or immigration status • institutionalization • stress (e.g., Perceived Stress Scale)^94^ • support • loneliness • religiosity/spirituality	• segregation, ethnic enclaves^95^ • neighborhood disorder^96^ • percentage in [area] with “high needs” (generally children and elderly)
Physical environment	• perceived safety and crime • walkability • green space • neighborhood aesthetic • neighborhood pride	• crime • walkability score (index of population density, business density, street connectivity)^97^ • satellite-derived green space • pollution^98^ • climate (e.g., heat) • food environment • noise • blue space (water)
Healthcare access and quality	• health insurance status or type • number of recent visits • time or difficulty needed to schedule an appointment • distance to generalists or specialists • access to specific preventive services (e.g., mammography) or health-related services (e.g., dentistry) • healthcare discrimination • health literacy • ability to afford care	• hospitals *per capita* • provider density within health service area or county (Area Health Resource File) • median driving/public transportation time • density of facilities offering different services (diagnostics, screening, treatment) • insurance coverage (census)
Combination measures	• proposed “polysocial”^99^^,^^100^ or “polyexposure”^101^ scores	• Townsend Deprivation Index^102^ • Social Deprivation Index^103^^,^^104^ • Social Vulnerability Index^105^^,^^106^ • Neighborhood Deprivation Index^107^ • Area Deprivation Index^108^ • Yost Index^109^ • AHRQ SES score^110^

### Socioeconomic domain

Socioeconomic measures are by far the most commonly reported SDoH and often include educational attainment and income, with employment status (or occupational prestige), net worth, access to government benefits, home or car ownership, food and housing security, and debt balance sometimes assessed within this domain as well. Of the most commonly assessed measures, educational attainment has the benefits of flexible modeling (e.g., continuous years of education vs. well-recognized thresholds of educational attainment such as a high school degree), largely being stable throughout the adult lifespan, and associating with social capital in a similar way across geographies. Income may also be modeled continuously or categorically (albeit with less well-recognized categories, which may vary across survey instruments); however, income varies throughout the lifespan, does not always reflect underlying socioeconomic status well (e.g., home-makers and retirees), and often has high levels of non-random missingness^111^^,^^112^^,^^113^ (e.g., missing in 20% of survey responders in the *All of Us* Research Program, with non-random missingness associated with demographic and other SDoH factors).

When examining area-level measures of socioeconomic status, it is possible to use single-factor measures that capture domains similar to those used for individual-level SDoH assessment (e.g., median household income). However, multiple complex deprivation indices or scores have been developed, generally leveraging data from the American Community Survey in the US, and are commonly used in clinical research.^102^^,^^103^^,^^106^^,^^107^^,^^108^^,^^109^^,^^110^ While these deprivation scores capture more diverse elements of SDoH, they may be less intuitive or interpretable than more simple measures, may be difficult to compare across geographies and time periods, and may in some cases include demographic data, thus complicating modeling with population descriptors.^114^ Lastly, these scores only modestly correlate with one another and perform variably in different settings and health outcome studies.^115^^,^^116^^,^^117^^,^^118^^,^^119^^,^^120^^,^^121^ There is no consensus within the field regarding which indices are most valuable in different research settings^114^^,^^115^^,^^122^ or even whether these more complex indices are superior to less complex, single-factor measures.

### Sociocultural domain

In the sociocultural domain, individual-level measures may include family and social group cohesion (using measures of social context such as the Berkman-Syme Index^123^), caregiver burden,^124^ social engagement (e.g., in religious^125^ or recreational activities), experiences of discrimination,^126^ and levels of institutionalization (e.g., incarceration). For studies examining racial and ethnic variation, questions about preferred language, parental ethnicity, nationality, place of birth, and immigration status may provide further information about cultural variation.

Area-level measures of sociocultural phenomena are less commonly used, as these elements are often subjective to the individual. However, the American Community Survey includes information on household makeup (e.g., single parent families), which is included in some deprivation indices, and ethnic enclaves have been defined based on geographical concentration of socioculturally homogeneous groups.^127^^,^^128^ Evidence for structural racism, the effects of sociopolitical systems that direct resources in ways that advantage certain groups and disadvantage others based on race or ethnicity, may also be reported at the area level, often proxied by measures of racial and economic segregation.^129^^,^^130^^,^^131^^,^^132^^,^^133^

### Physical environment domain

SDoH in the physical environment are often but not always measured at the area level. However, some studies assess individual-level measures, often by querying participant perception of their physical environments, including perceptions of safety and crime, walkability, green space, neighborhood aesthetic, and neighborhood pride or cohesion.^134^ Others assess physical exposures indirectly by examining proxies such as biological sequelae of exposures (e.g., blood or urine levels of heavy metals).

By contrast, area-level measures of the physical environment are common and include measures of housing age and quality (also sometimes considered a socioeconomic measure), public space and resources (e.g., food environment, green space), climate (e.g., extreme heat), and air pollution.^135^^,^^136^ Many of these factors are strongly associated with both socioeconomic status and with racial and ethnic makeup of neighborhoods, a phenomenon linked to historical practices of structural racism such as redlining.^137^ For example, sources of the most commonly measured and regulated air pollutants are often concentrated in impoverished, urban communities, particularly those impacted by segregation and other forms of structural racism.^138^^,^^139^

### Healthcare access domain

Individual-level healthcare access is most commonly proxied by health insurance status or type, another measure that is variably assessed in different datasets (e.g., binary insured status vs. complex insurance type variables) and can be difficult to categorize consistently. Other measures may include the number of recent generalist or specialist visits, difficulty of scheduling appointments, distance to physicians or hospitals, access to specific preventive (e.g., mammography) or health-related services (e.g., dentistry), or acceptability of health-system encounters.^140^ With increasing use of electronic health record databases to capture clinical encounters and treatment, additional measures of healthcare access, utilization, and quality, such as depth of the medical record, may be more readily incorporated into existing studies, albeit with limited applicability for individuals with fragmented care across multiple health systems.^141^

Area-level healthcare access may be measured by examining concentration of medical services, including primary care, specialty care, allied health services (e.g., dentistry), and critical access hospitals.^142^ Large-scale measures of insurance coverage (e.g., state-level expansion of Medicaid services under the Affordable Care Act) have also been used as measures of healthcare access at the area level.^143^

### Harmonization of SDoH measures across diverse datasets and contexts

Harmonization of SDoH across datasets represents an enormous opportunity to increase the power and generalizability of findings related to genetic and environmental interactions across populations. However, in many cases, the complexity of SDoH contexts, domains, measures, levels of assessment, and variable transformation may result in two studies having no exact overlap of SDoH variables. Even when an equivalent variable with similar transformation exists between datasets, the canonical epidemiological concepts of “populations, place, and time”^144^ are essential study design considerations related to the comparability of analytic findings. Specifically, replicability of SDoH-related analyses across datasets may be limited by the variable meaning and impact of an SDoH measure across place and time (Figure 4), including timing of exposure across the lifespan, with implications for validity and interpretation.^145^

When these aspects have been considered and deemed comparable between datasets, the following considerations may aid the researcher to assess validity and appropriateness of SDoH measure selection and comparison across cohorts.

### Considerations for harmonization of individual-level SDoH

Given the broad range of SDoH measures that may be of interest, it is common for studies not only to capture different measures but also to query and transform responses in different ways, creating challenges with pooling measures of SDoH across datasets.^146^ For example, family income may be captured as continuous dollars, variable categories of income, or a calculated value such as the income-to-poverty ratio, which accounts for household size as well as income. Even measures assessed and transformed identically may not be directly comparable in populations living in different contexts (e.g., urban vs. rural) or at different points in time (e.g., between generations), as the health impact of any SDoH depends on societal context (Figures 4A and 4C), and differences in distribution of SDoH between cohorts may impact analytical results (Figure 5). This limitation is sometimes addressed by adjusting for inflation (for income), including a secular time trend measure as a covariate or interaction term in models, or normalizing within a geographic context or to a construct such as the consumer price index,^147^ although methods are not applied uniformly across studies.

Regarding timing of exposure, most large epidemiological studies recruit middle-aged individuals, so reported SDoH generally reflect adult SDoH.^148^ Questions asked about childhood and parental SDoH can enable evaluation of earlier life influences of these factors on health,^149^ albeit with risk for recall bias, and certain multigenerational epidemiological cohorts may allow investigation of life course SDoH exposures (e.g., Growing up Today Study, Framingham Offspring Study, Project Viva).^150^^,^^151^^,^^152^

Finally, high levels of missingness of self-reported SDoH variables are common,^153^ often in observable patterns (i.e., “missing at random,” such as decreased reporting of income in those with lower educational attainment) but not always (i.e., missing not at random, such as decreased reporting of income among those with low income),^153^ such that different studies may have distinct patterns of informative missingness (Figure 4B). Rates of missing data for key variables should be examined in every dataset, and sensitivity analyses employing restriction, imputation, or scenarios with various degrees of informative missingness may be beneficial to understand the potential impact of missing SDoH data.^154^^,^^155^

Because of these factors, it is often impossible to exactly replicate an analysis involving individual-level SDoH across datasets, particularly in legacy cohorts. Newer studies, such as *All of Us*, have been designed with harmonization of SDoH measures in mind; however, there are still important considerations related to non-random missingness of SDoH measures, with White individuals and those with higher educational attainment and literacy more likely to complete SDoH and health surveys.^26^ Consortia and national research entities have been established to harmonize phenotypes,^91^^,^^156^^,^^157^ including SDoH, across datasets, but these efforts are ongoing.

### Considerations for harmonization of area-level SDoH

Geospatially derived SDoH are susceptible to measurement error when used as proxies for individual-level SDoH^120^ and generally should be interpreted as area-level exposures rather than individual-level SDoH (“the ecological fallacy”).^158^ However, individual- and neighborhood-level SDoH exert distinct influences on health, and both may be relevant to particular research questions.^159^^,^^160^^,^^161^ Careful consideration of whether area-level SDoH is an appropriate measure, either alone or in combination with individual-level measures, given the conceptual framework, hypothesis, and intended interpretation of the study is required prior to selecting these measures.

However, when appropriate, neighborhood-level measures have many advantages from a design and data harmonization perspective as they can be assessed using the same data source (e.g., decennial and multiyear US Census and American Community Survey databases),^162^ at the same spatial scale, using the same analytic pipelines, for participants across multiple studies when participant-level geographical information is available.^163^ Neighborhood databases often capture information going back decades; for example, the US census captures population socioeconomic characteristics at different spatial scales for linkages back to the 1980s and earlier. Given this, area-level SDoH measures may be retroactively assigned to participants in legacy cohorts, and studies with data on residential histories collected over successive questionnaire cycles may allow for the investigation of time-varying influences of area-level SDoH.^164^^,^^165^^,^^166^

Choice of area-level measure requires consideration and should arise from evidence-based conceptual frameworks associating SDoH with the outcome of interest. Multi-measure indices can be useful if the goal of including SDoH is to statistically control for multiple potential influences of non-genetic social environmental factors that correlate with race and ethnicity. However, if a specific gene-environment interaction is of interest or if a study is designed to infer causality, it is preferable to use the individual measures that best capture the association of interest. If using a deprivation index, the features contributing to the index should be carefully examined to ensure they are appropriate to the study question and statistical analysis plan and do not unintentionally introduce errors such as collider bias.

When using area-level SDoH measures, it is critical to consider the spatial scale of the analysis,^161^^,^^167^^,^^168^^,^^169^ as the choice of spatial scale may determine the strength and sometimes even the direction of association.^120^^,^^170^ In general, larger spatial scales are associated with less significant variability for physical environment measures such as air pollution^171^^,^^172^ but more significant variability for socioeconomic measures; in particular, the associations of more granular census tract- and census block group-derived SDoH are more consistent than associations reported at the county or ZIP code.^120^^,^^170^ In many datasets, privacy concerns limit geographical information to ZIP code or even three-digit ZIP code as the smallest geographic identifier, although geomasking procedures may allow for balance of privacy concerns with the need for sufficient precision to rigorously study important geospatially derived SDoH.^173^^,^^174^

In summary, the application of SDoH in biological and clinical research is an evolving field with many possible measures, many of which are interrelated and that are often captured differently in different datasets or not at all. We recommend selection of measures based on conceptual frameworks grounded in prior research, with consideration of appropriateness of the measure to the study question, thoughtful harmonization across studies conducted in different places and times, and cautious interpretation of results, recognizing that many SDoH variables act as proxies for intangible features of social position and lived experience (Table 2).

**Table 2:** Best practices and ethical considerations in the design, implementation, and interpretation of analyses examining the intersection of PRS and SDoH on health outcomes across diverse populations

**Design Question**	**Best practices**	**Ethical considerations**
What questions can be answered using genetics and SDoH in clinical studies?	Analyses incorporating PRS and SDoH may be undertaken with the overarching goals of effect estimation (explanation) or prediction. Both of these goals require careful consideration of how each variable is characterized in the model, based on a well-articulated conceptual understanding of the hypothesized role of the variable (e.g., an independent effect, effect modification, or mediation), which motivates the statistical analysis plan and modeling specifications	While inclusion of population descriptors and SDoH in explanatory models may be appropriate, results should be interpreted cautiously in order to prevent causal interpretations for variables that, in many cases, act as proxies for true latent risk factors
How should I select my PRS? What implications does this choice have on my analysis?	Selection of a PRS for further characterization with SDoH should focus on whether the PRS is appropriate for the individuals and/or populations within the target study (i.e., has transferability been previously demonstrated for this PRS?). For studies implemented on defined populations, there may exist population-specific PRSs that have been constructed, evaluated, and calibrated for that population. If a multi-ancestry or ancestry-agnostic PRS is needed, there may exist a PRS constructed using diverse GWAS data and/or methods specifically designed to leverage multiple populations. For more details on the construction of a PRS, see Kachuri et al.^157^	Most PRSs have been developed in populations of European ancestry and with favorable SDoH distributions. Application to populations with different profiles of genetic, SDoH, or both factors is known to result in decreased predictive accuracy of PRSs^9^^,^^68^
How should I describe my study populations?	Common population descriptors include social constructs (race, ethnicity), categorical genetic ancestry, and continuous or categorized genetic similarity. Unless the study question specifically relates to the health disparities arising from race- or ethnicity-based discrimination (e.g., structural racism) for which self-reported or societally assigned racial or ethnic identity may be the most appropriate exposure, genetic similarity is generally preferred in order to avoid typological thinking and the misinterpretation of race or ethnicity as causal elements of disease rather than social constructs.^30^ When either descriptor may be appropriate (e.g., in analyses examining PRS-SDoH interactions), the correct choice of population descriptor may depend on the intended interpretation or application of the study results	Assignment of individuals into race- or ancestry-based categories creates artificial divisions across the spectrum of human diversity and may lead to entrenchment of historical race- and nationality-based biases
What SDoH measure should I use?	SDoH measure selection should be based on a well-considered causal framework that is specific to the study question and grounded in prior research When available, individual-level measures are generally preferred over area-level measures,^120^^,^^175^^,^^176^^,^^177^ although modeling of both individual- and area-level metrics may be warranted and may provide a more nuanced understanding of the breadth of SDoH risk factors Ability to harmonize across datasets^91^ may be a consideration in measure selection but should not supersede selection based on thoughtful evaluation of the hypothesized causal framework	Findings related to SDoH should be interpreted cautiously, as any single SDoH factor may act as a proxy for other SDoH factors. Sensitivity analyses examining the impact of highly correlated variables may help to elucidate underlying relationships
How should I assess SDoH measures?	SDoH are often self-reported with high levels of missingness. Prior to beginning analyses, researchers should examine each measure for informative missingness^111^^,^^113^^,^^153^ Examine the relationship of the SDoH measure with the outcome (e.g., linear, threshold effect) prior to modeling to ensure appropriate model specification. *A priori* knowledge of the relationship of the measure with social position should guide variable transformation when available (e.g., degree thresholds for educational attainment)^146^	Consider how exclusion or transformation of missing data may lead to selection bias or alter results of an analysis
How can I harmonize SDoH across studies, or replicate my SDoH-related findings in a similar cohort?	Harmonization to gold-standard measures examined previously in the field is ideal whenever possible. Both the choice of measure (e.g., choosing an education-related measure in both datasets) and any transformation of the measure (e.g., categorizing continuous measures into categories matched to another dataset) should endeavor to support reproducibility with similar studies in the field. In addition to choice of measure, both the place and time (both historical period and stage of life) of the exposure should be consider to ensure comparability across datasets^115^^,^^116^^,^^117^^,^^118^^,^^119^	Harmonizing SDoH measures without careful attention—for example, merging absolute income categories drawn from different time periods—may introduce false equivalencies and obscure findings in underrepresented populations. It is essential to ensure that harmonization efforts do not result in exclusion of underrepresented groups; incomplete data for a particular group in one cohort should not justify their wholesale exclusion from cross-cohort analyses
I want to use an area-level SDoH measure. What should I consider?	More granular area-level spatial scales (e.g., at the census tract level) are preferred over less granular spatial scales (e.g., at the county level) for most SDoH,^115^^,^^120^^,^^178^ although measures of the physical environment or sociopolitical determinants of health may be appropriate at larger spatial scales^171^^,^^172^ More complex measures are not clearly superior to less complex, more interpretable measures, and SDoH indices have limited agreement between one another^115^^,^^116^^,^^117^^,^^119^^,^^120^^,^^175^^,^^176^^,^^177^ In addition to spatial resolution, consider the time period during which the genetic and SDoH exposures are most relevant to the disease of interest (e.g., cancer may require a longer time period than other chronic diseases).	Correct interpretation of area-level SDoH measures should avoid the “ecological fallacy” by attributing the risk factor to the community and not to the individual (e.g., individuals living in areas with low median income have higher risk of mortality and not individuals with low income have higher risk of mortality) Consider how findings may be interpreted and used to allocate scarce resources for public health and development
How should I interpret my study results?	Many SDoH variables act as proxies for intangible features of social status and lived experience, and it is critical to employ conceptual frameworks that capture the hypothesized role of SDoH within a research study When considering the generalizability of study findings, it may be helpful to compare the distribution of SDoH in the study setting to the distribution in the population to which results may be applied	Consider how results may be interpreted, both in the scientific literature and in the lay literature, to ensure that findings will not be used to further disadvantage any population studied. It is also critical to consider the impact of any results should they be implemented in clinical practice (e.g., disease prediction algorithms)
What features should I examine prior to analysis of genetic and SDoH data across diverse populations?	The distribution of genetic and SDoH instruments should be examined in each population under study. If SDoH or other contextual factors differ significantly between populations, context-specific calibration of PRS, in addition to calibration for genetic similarity, may be appropriate^68^ It may also be important to examine the performance of each genetic and SDoH instrument in each population studied or to examine instrument-by-population interactions to ensure validity of analysis results, particularly in underrepresented groups	Ensure adequate representation of each population group under study; if any group(s) are underpowered for the outcome of study, it may not be appropriate to interpret results in that group. In this scenario, ongoing advocacy for increased diversity of genetic datasets (from both a genetic and SDoH perspective) will be critical to improve the scientific community’s ability to make inferences and predict outcomes for groups underrepresented in medical research

### Analytical considerations for integrating PRSs and SDoH across populations

Multivariable modeling of a PRS with SDoH variables requires clear analytical goals that determine model structure. These goals broadly fall into two main categories: effect estimation (i.e., explanation) and risk prediction, each with important implications for health equity. The consideration of both genetic and SDoH risk factors in explanatory analyses can improve our understanding of true factors underlying adverse outcomes, often refuting discriminatory beliefs such as racial essentialism. Similarly, methods adjusting for non-genetic contextual elements, including SDoH, have been shown to reduce miscalibration of PRS in populations poorly represented in the discovery cohort,^68^ with potential applications to improve PRS accuracy and disease prediction in population groups underrepresented in research, and particularly those underrepresented in genetic research.

However, regardless of the primary goal of effect estimation or prediction, any planned statistical modeling requires appropriate characterization of the role of PRS and SDoH within the well-articulated conceptual framework of the study (for example, as an independent exposure [main effect], effect modifier, or mediator).

### Modeling of PRS and SDoH: Effect estimation

Main-effect estimation requires (1) the consideration of temporality between variables included in the model and (2) inclusion of variables that remove confounding but do not introduce bias through over-adjustment.^179^ In the context of effect estimation, the set of variables selected for adjustment is dependent on the primary effect for estimation (e.g., PRS or SDoH,) and should derive from a conceptual framework based on established relationships between risk factors and disease, allowing for differences between populations, if appropriate (e.g., Figure 6).

In the context of effect estimation for disease risk, a PRS measures a set of germline genetic variants that precede all other variables, leaving population stratification and cryptic relatedness as the only major potential confounders for adjustment (Figure 6).^180^ For example, any SDoH variable, such as access to preventive care, cannot temporally precede germline variation assigned at birth. This is the same premise on which Mendelian randomization is based.^181^ However, confounding by SDoH may still be introduced through (1) selection bias and (2) residual population stratification. Selection bias may occur when relationships exist between participation or inclusion, SDoH, and germline variation, as in a situation where genetic ancestry is related to PRS (or PRS performance) and SDoH, which also influence either study participation or completeness of data (Figure 4B). In case-control designs, correction for this bias may not be feasible through adjustment due to collider bias.^182^ Bias from residual population stratification may also require the additional adjustment for SDoH for the correct estimation of a PRS effect. For example, if estimating the causal effect of a PRS for diabetes and T2D incidence (Figure 6), adjustment for population stratification (impacting both the PRS and the distribution of SDoH) as well as SDoH themselves may be required to ensure accuracy of effect estimation. Indirect relationships through parental genetics and parental SDoH, each influenced by upstream historical processes that also relate to population admixture and therefore PRS, may result in biased effect estimation. For example, within the multiethnic cohort there is evidence of genetic similarity (via principal components) being correlated with neighborhood SES not only across all self-identified race and ethnicity populations but correlated within each population as well.^51^^,^^183^ Similar findings have been seen in the UK Biobank.^184^^,^^185^

Effect modification, or effect heterogeneity, occurs when the effect of the primary exposure varies across strata of another variable.^186^ For example, in Figure 4C, the magnitude and direction association of SDoH with T2D prevalence varies by population; in Figure 6, effect modification would exist if the marginal effect of the PRS was observed to depend on an individual’s SDoH (as depicted in Figure 3, path 2 with the marginal effect represented as the difference between the high and low PRS at each level of the SDoH distribution). Effect modification is commonly tested using regression models with an interaction or product term between the exposure and proposed modifying variable, specifically assessing the interaction on the multiplicative scale, or differences in *relative* risk. This form of interaction may also be assessed as the product of risks. On the additive scale, interaction may be assessed through comparison of differences in *absolute* risk, or risk difference, across a modifying variable.^187^ In most cases, additive interactions reflect differences in underlying absolute risks, or population-level impact of a risk factor, between populations with different distributions of the modifying variable, rather than true differences in underlying biology (Figure 5). If there is no effect modification on the multiplicative or relative scale, then an additive interaction will be present so long as absolute risk varies for different levels of the modifier.

While specific single-nucleotide polymorphism (SNP)-SDoH or SNP-environment interactions can be difficult to detect, particularly when using genome-wide significance thresholds, the increasing size of available genetic datasets and novel methods to detect interaction signals have improved the ability to detect and interpret these interactions.^188^^,^^189^^,^^190^ In contrast, the investigation of PRS-SDoH interactions may allow greater power by reducing the number of statistical tests evaluated to only the PRS in combination with the SDoH variables. In addition, this strategy may improve interpretability by identifying subgroups of individuals defined by PRS strata in which SDoH variables may have differential effects or vice versa. As an illustration, Norland et al. assessed the influence of PRS and 22 SDoH—primarily related to income, employment, and food or housing security—on coronary heart disease (CHD) risk in the All of Us cohort, exploring interactions between a pooled individual SDoH score and PRS and providing researchers with a useful example of how SDoH burden can be modeled in interaction with genetic risk.^191^

As discussed above, an important consideration related to effect modification in genetic studies is transferability, or whether a PRS will have similar associations or predictive value in populations with different underlying population structure and SDoH distributions (Figure 3). To evaluate transferability, PRSs are assessed across samples from different populations, and in many cases the association of the PRS with outcomes, or its predictive value, may decline in populations with low genetic similarity to the derivation cohort,^60^ related to differences in genetic architecture, such as LD structure and allele frequencies. However, PRS performance can also vary even within assumed genetically homogenous populations, which may be attributable to other contextual variables, including SDoH (Figure 3, path 2).^184^^,^^192^ While it may appear in this scenario that effect modification of the PRS exists across populations, the variation in effect sizes may be due to a failure to properly incorporate differences in the prevalence of effect modifiers that are associated with both the PRS and disease. For example, while there may be a single true PRS effect across all populations, if the prevalence of the modifier differs by population, the estimated marginal PRS effect for disease risk at the same mean PRS may differ across populations (Figure 3) due to the presence of an interaction with PRS. In Figure 3, path 2, in the presence of modification, we see population 2 and 3 have a smaller difference in risk explained by the PRS due to greater modifier prevalence, which interacts with the PRS; in this scenario, adverse SDoH may increase the risk of disease even in individuals at low genetic risk, thus resulting in higher overall disease prevalence and less variability explained by the PRS. Alternatively, under the scenario of no modification, the population-specific modifier distribution would not affect the population differences in risk. While it has become a common practice in PRS assessment to calibrate based on genetic similarity, recent efforts to incorporate potential contextual modifiers such as SDoH have allowed researchers to ascribe variation that used to be attributed to genetic similarity to SDoH causes. For example, PRSs have generally been constructed in socially advantaged populations, resulting in less accurate risk estimates in socially disadvantaged populations. Methods that calibrate PRS across non-genetic contextual factors have been shown to improve PRS performance.^68^

Lastly, mediators are factors that are at least partially caused by the exposure and that lead to the outcome, acting as “a step in the causal chain.”^187^ Mediation analysis allows for decomposition of direct and indirect (or mediated) effects of an exposure, and therefore calculation of the proportion of the effect mediated through a given mediator.^193^^,^^194^ Correct statistical estimation relies on strong assumptions about the completeness of the causal framework, including the assumptions of no residual confounding in the exposure-outcome or mediator-outcome relationships.^186^^,^^194^ Mediation analysis may be appropriate when SDoH are hypothesized to act as mediating—or mediating and modifying—factors within the conceptual framework of the study, or when another variable is thought to mediate the effects of PRS and/or SDoH on health outcomes (e.g., BMI or insulin resistance may mediate the association between SDoH and T2D in Figure 6).^193^ Even when SDoH is not considered as a key exposure or mediator, adjustment for SDoH may address confounding by capturing environmental factors related to historical processes resulting in modern population stratification. When performed correctly, these analyses may inform biological mechanisms underlying PRS-SDoH-disease pathways, quantify the extent to which population-level inequalities would persist if SDoH-related variables were similar between populations, or estimate the proportion of the effect of immutable polygenic risk on the outcome that could be modifiable given an intervention on a specific SDoH.^35^

### Modeling of PRS and SDoH: Prediction

Regarding prediction, models that incorporate all relevant risk factors are expected to explain greater variability than those incorporating fewer components of risk, and multiple studies have demonstrated independent effects of genetic and SDoH factors in predicting disease.^23^^,^^100^^,^^101^^,^^191^^,^^195^^,^^196^^,^^197^ Although neither PRS nor SDoH are routinely used for risk prediction in routine clinical care, there are evolving efforts to include both factors.^198^^,^^199^ The personal and public health impacts of implementing PRS- or SDoH-based scores may be optimized if both factors are considered together but only if based on well-specified and well-calibrated models. As in effect estimation, it is critical not only to include all relevant risk factors but to properly specify them in the model based on the hypothesized conceptual framework of the study. For example, expanding joint models with the inclusion of PRS-SDoH interactions may be helpful to improve predictive accuracy of disease risk models, advance precision medicine by identifying high-risk individuals or groups, and even to inform our understanding of biological pathways and/or mechanics in which SDoH factors are most impactful.

Often, for population and clinical translation, risk prediction models are constructed on the absolute-risk scale and thus rely heavily on the specification of baseline risk.^200^ As baseline risk often varies by population (as in Figure 5), careful incorporation of population descriptors as variables or calibration of baseline risk needs to be considered.^201^ To properly explain risk in a population, models must include all variables that describe risk within each subpopulation. If aiming to model the population-level disparities that contribute to disease risk, it is important to construct statistical models that clearly delineate groups of individuals with risk factors that differ in their distributions while also recognizing that these risk factors may be correlated with unknown factors or reflect broader systemic determinants.

Diverse cohorts can be used to examine how the distribution differences of known non-genetic risk factors might explain population differences in either incidence or patterns of population attributable risk.^183^^,^^202^^,^^203^ However, it is unclear to what extent genetic risk may relate to observed differences. Similar approaches attempting to describe risk differences based on the distribution may have challenges as the observed PRS distributions are potentially confounded by genetic ancestry and are thus often standardized by population descriptor variables before evaluation.^204^ However, recent studies have attempted to assess the genetic basis for population differences either by directly comparing the PRS distributions while considering the genetic architecture^205^ or by examining genetic clusters estimated from PRS-related variants across multiple closely related traits to provide insights into ancestry-associated differences in risk.^14^

### Ethical considerations

Due to long-standing race- and class-based discrimination throughout the world, the integration of both population descriptors and SDoH in scientific analyses requires careful consideration of the biases, interpretation, and possible implications of the research conclusions, both in the scientific and lay communities (Table 2). Although these concerns may be relevant in myriad scenarios, we highlight the importance of these considerations in study design and interpretation.

First, in any study reporting on the impact of population descriptors or SDoH on health, it is critical to identify study limitations that may invalidate conclusions drawn for one or more groups. This includes examination for adequate representation and selection bias. For the former, it is important to consider the sample size of each included group, including cross-tabulation of key populations and exposures, to ensure that the study is powered to comment on findings in that group. For example, the UK Biobank includes approximately half a million participants, but fewer than 5% of these identify as a race or ethnicity other than White.^28^ When stratifying by other factors, such as environmental exposures, or when studying rare outcomes, it is unlikely that there is always sufficient representation of these groups to draw firm conclusions (i.e., nonpositivity^206^). Regarding selection bias, it is important to consider whether enrolled subjects, particularly those underrepresented in medical research, are representative of the underlying population. For example, among All of Us survey responders, greater than 90% of participants who identify as Asian have at least some college education, and greater than 40% have an advanced degree, significantly higher than among Asian-identifying individuals in the general population.^207^ In this scenario, findings within this group may not be representative of other Asian-identifying populations in the US.

Second, researchers must use caution when using study designs or language that imply causality, including mediation analyses. Although conceptual frameworks should always be employed to guide analyses relating to sociocultural phenomena, these frameworks often rely on hypothesized relationships that may be incomplete, heterogeneous across populations, or even incorrect. Both race and ethnicity (and genetic ancestry by association) and SDoH measures are often employed as variables that may act as proxies for complex sociocultural phenomena that cannot be simplified into measurable variables. As mentioned previously in this review, many researchers caution against using SDoH without proper specification, or at all, due to the risk of oversimplifying complex social dynamics and introducing bias if SDoH is not accurately measured or appropriately modeled. As such, associations observed between population descriptors, SDoH, and health outcomes should be interpreted cautiously, with causal language reserved for studies with very strong causal frameworks or study design (e.g., randomized trials with interventions impacting SDoH such as the Moving to Opportunity Study).^208^

Finally, it is critical to consider how study findings may be interpreted, not only by academic readers but also by the lay public, and what implications these findings may have for minoritized communities. As race, ethnicity, ancestry, and SDoH are frequently stigmatized, findings that associate these factors with adverse outcomes or stigmatized behaviors may unintentionally lead to the entrenchment of previously held biases. Research findings may directly impact clinical care as well. There is a growing movement to identify bias introduced by the use of racial and ethnic designations in clinical algorithms^36^^,^^209^^,^^210^^,^^211^^,^^212^^,^^213^ and to propose alternate measures if possible.^214^^,^^215^ However, SDoH have been considered differently, and some authors have suggested including measures of SDoH in clinical calculators.^199^^,^^216^ Although the intent of these authors is to capture SDoH-related variability associated with health disparities without anchoring on the social construct of race and ethnicity, the downstream effects this may have on individuals and communities have not been studied. Moreover, clinical prediction models derived from real-world datasets may unintentionally incorporate biases and inequities in healthcare, thus resulting in prediction models that perpetuate underlying health disparities. Careful consideration of how research findings may impact clinical care and health equity are critical prior to implementing these findings. Thoughtful and humble engagement with members of potentially affected communities may help to guide interpretation and dissemination of results.^217^

## Conclusions and future perspective

In sum, growing awareness of the concomitant impacts of genetic and SDoH risk factors for disease has led to broad interest in incorporating these factors into both explanatory and predictive models of health and disease. However, many nuances of the distribution, measurement, and analysis of these factors across diverse populations living in diverse geographical, physical, and sociocultural contexts complicates these analyses. Each of these considerations, as well as the implications of study findings on society and health, particularly for minoritized groups, is critical for all analyses jointly examining PRS and SDoH.

As the field continues to grapple with these complexities, it is also poised to address them. Concerted efforts are already underway to create polygenic scores that have high predictive value across datasets. These efforts leverage large consortia with access to biobanks and longitudinal cohorts with increasing diversity improving power, rigor, and generalizability of findings. In addition, advances in statistical approaches better capture and model genetic associations with disease across diverse genomes. Similarly, expert teams are working to harmonize key phenotypes, including SDoH, across cohorts. The study of SDoH will benefit from growing interest in the routine and standardized collection of complex individual-level SDoH measures, and efforts to ensure complete data across population groups may assist to prevent selection bias in future analyses. Meanwhile, the robust availability of area-level SDoH measures and indices may be harnessed to model disease risk more specifically, characterize key environmental contributors to specific health outcomes, and identify geographic areas that may benefit from additional resources. Lastly, and perhaps most importantly, national and international goals to improve the inclusion of diverse individuals in all types of research will result in more representative datasets that capture greater genetic and environmental variation in the human experience and empower data scientists for discovery. The field must continue these robust and ongoing efforts improving representation, harmonization, modeling, and analytic methods development. This will move research in this area toward a more equitable and scientifically rigorous understanding of the interplay of genetic and environmental risk within and across populations, ultimately enabling more accurate disease predictions and more just applications of precision medicine.

## Date and code availability

This study did not generate or analyze datasets.

## Acknowledgments

On behalf of the PRIMED Consortium SDoH Working Group (https://primedconsortium.org/). S.J.C. is supported by the American Diabetes Association (7-21-JDFM-005). H.S.I. is supported by 10.13039/100000066NIEHS K01ES035734. J.L.S. is supported by HL07111-45. D.V.C. is supported by U01CA164973. This publication was supported by the 10.13039/100000002National Institutes of Health for the project “Polygenic Risk Methods in Diverse Populations (PRIMED) Consortium,” with grant funding for study sites CARDINAL (U01HG011717), CAPE (U01HG011715), D-PRISM (U01HG011723), EPIC-PRS (U01HG011720), FFAIRR-PRS (U01HG011719), PRIMED-Cancer (U01CA261339), PREVENT (U01HG011710), and the Coordinating Center (U01HG011697). The content is solely the responsibility of the authors and does not necessarily represent the official views of the National Institutes of Health.

## Declaration of interests

S.J.C. reports employment of a family member by Depuy-Synthes.
